# Burden experienced by community health volunteers in Taiwan: a survey

**DOI:** 10.1186/1471-2458-13-491

**Published:** 2013-05-21

**Authors:** Yueh-Mei Gau, Petra Buettner, Kim Usher, Lee Stewart

**Affiliations:** 1 School of Nursing, Midwifery & Nutrition, James Cook University, Townsville, QLD 4810, Australia; 2 Department of Nursing, Chang Gung University of Science and Technology, Taoyuan, Taiwan; 3 School of Public Health, Tropical medicine & Rehabilitation Sciences James Cook University, Townsville, QLD 4810, Australia; 4 School of Nursing, Midwifery & Nutrition James Cook University, Cairns, Australia

**Keywords:** Burden, Volunteers, Community health volunteers, Health promotion

## Abstract

**Background:**

Volunteers in Taiwan complement the delivery of health services by paid health professionals. However, in doing so, community health volunteers experience burdens associated with their activities. The reasons for these burdens and degree to which they are experienced are explored in this paper. Our study adds to international research regarding the burden experienced by volunteers. This project is the first to assess how community health volunteers in Taiwan experience burden.

**Methods:**

The 20 item Burden on Community Health Volunteer (BCHV) instrument, specifically designed for this project, was administered to 435 volunteers attached to Community Health Promotion Development Centres in northern Taiwan.

**Results:**

The overall burden experienced by volunteers is relatively low. However, a multivariate adjusted regression analysis revealed significant differences in volunteer burden depending on the number of people each volunteer served on average per week, as well as the volunteer’s marital status and their perceptions about personal health. Volunteers who served many people and who perceived their own health as poor experienced a higher level of burden. Those who were a widow or a widower felt less burdened than others.

**Conclusions:**

The results of the study identify areas where burden is high and where strategies can be developed to reduce the level of burden experienced by community health volunteers in Taiwan. Community health volunteers in Taiwan complement the role of nurses and other health care providers so their retention is important to ongoing service delivery.

## Background

Community health volunteers (CHVs) in Taiwan assist with the delivery of health promotion and monitoring activities across the community, contributing significantly to the health workforce and service delivery. As unpaid workers, their role is extremely important to the ongoing delivery of community care to a rapidly aging population in Taiwan [1,2]. Volunteers are also used effectively to supplement the health workforce in many other countries [3-6] and, given the current constraints on health service delivery in many developed countries, offer a potential solution to health staff shortages. Currently, however, little is known about the burden experienced by these volunteers while carrying out their role, especially those undertaking this role in Taiwan. This paper presents the results of a study that measured the burden experienced by a sample of CHVs in northern Taiwan.

In Taiwan, approximately 300 Community Health Promotion Development Centres (CHPDCs) in cities and communities contribute to the healthy community-development programme funded by the national Department of Health [7]. The volunteers of each CHPDC are educated to assist residents by delivering health promotion and health monitoring activities. The CHVs are important to the delivery of health services because they help to diffuse health promotion in their local community and complement the paid workforce in the area of health monitoring [7-10]. In particular, their role includes working with people with a disability, the elderly, and women and children. Community health volunteers are also expected to visit families for the purpose of monitoring health. For example, they provide blood pressure measurement for older community residents, and conduct health behaviour change programs such as cessation of smoking and betel nut chewing [7].

To become a CHV, a person must be nominated by the leader of a local community association and attend a volunteer training course. After completing the course, volunteer duties are determined by the leader of the centre. The CHVs, like other community health workers, are expected to maintain and promote the health of the community [11]. In Taiwan there are three levels of CHVs. The ‘fundamental’ volunteers are the newest volunteers, those who have just begun in the role. ‘Cadre’ level volunteers are more experienced and capable. ‘Leaders’ are the most experienced volunteers; all volunteers undertake health promotion activities in the community while the ‘leaders’ also oversee the work of other volunteers. Volunteer services are overseen by the head of the CHPDC (known as the Chief). All ‘Chiefs’ are volunteers for the program. Some are also chiefs of a neighbourhood or a village. Chiefs are in charge of the community health promotion development program, they work with other volunteers and leaders in the community, organise volunteer teams, and plan how to use the minimal funding received from the Government or community for health promotion activities.

Burden, an accepted component of burnout, can be avoided or ameliorated if detected early. Most research on volunteer burden to date has focused on the burden experienced by family care givers in the community, especially of the elderly with dementia. It has been suggested that non-family volunteers are affected by the same type of negative impacts, or burdens, as for example, those experienced by family carer givers [12-14]. However, previous research on burden experienced by community health volunteers in countries other than Taiwan identified the following: work related to the physical and emotional nature of the role, the time taken to undertake the activity, insufficient emotional support from family, other volunteers, community health nurses and residents, lack of instrumental support, poor communication skills, lack of cooperation and lack of confidence [15-17].

### Study objective

The objective of this study was to examine the burden experienced by community health volunteers in Taiwan.

## Methods

### Study design

A cross-sectional survey was undertaken in 2010.

### Study locations, sampling procedure and recruitment of participants

**Figure 1:**
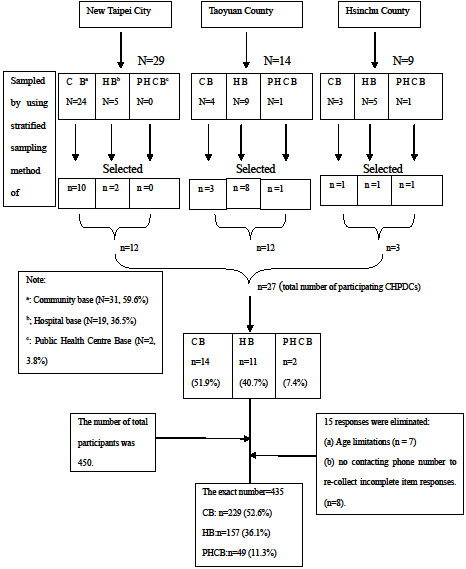
**Sampling of Community Health Promotion Development Centres (CHPDCs). **^a^CB = Community Base; ^b^HB = Hospital Base; ^c^PHCB = Public Health Centre Base.

The sample sites in New Taipei City, Taoyuan County and Hsinchu County, all located in northern Taiwan, were chosen because of their availability. There were 52 CHPDCs in the three municipalities at the time of the study. Of a total of 29 CHPDCs in New Taipei City, 14 in Taoyuan County, and 9 in Hsinchu County, 12, 12, and 3 respectively agreed to participate in the study. Of the participating CHPDCs, 14 were community, 11 were hospital and 2 were public health centre based (see Figure 1). The first author (Y-M.G) distributed the surveys through the head of the participating CHPDC and returned to collect completed surveys on a number of occasions.

The number of volunteers in each CHPDC varied between 20 and 50. The inclusion criteria were: (a) aged 20 years or older (the legal age of consent in Taiwan is 20); (b) volunteered for at least 3 months in the community health field in any type of CHPDC; and, (c) were involved in the delivery of health promotion activities to residents in the community. The number of total possible participants was 450. Fifteen responses were eliminated because of age limitations (n = 7) or due to surveys without a contact phone number that prevented the researcher from following up incomplete item responses (n = 8). Therefore, the final sample consisted of 435 volunteers (response rate = 97%) between the ages of 20 and 88; 229 (52.6%) from community based CHPDCs, 49 (11.3%) from public health CHPDCs, and 157 (36.1%) from hospital based CHPDCs. Figure 1 outlines the sampling of CHPDCs.

### Measures

The authors developed the Burden on Community Health Volunteers (BCHV) tool for the purpose of the study. The BCHV is a 20 item scale that uses a 5-point scale from never (1) = never occurred at any time, seldom (2) = occurred once a month, sometimes (3) = occurred once a week, often (4) = occurred twice a week, to always/nearly always (5) = occurred almost every time. A summative score of the 20 items was used; this results in a possible score for participants ranging from 20 to 100. The higher the score, the greater the volunteer’s perception of burden. The survey also included widely used demographic questions relating to factors associated with variations in personal conditions and volunteer experiences (see Tables 1 and 2).

**Table 1:** The demographic characteristic and information relating to the community health volunteer position of the survey participants (N = 435)

**Characteristic**	**Descriptive statistic**
% Female participants	84.3% (n = 366)
Mean age (±SD)* [years]	59.1 (±10.8)
% Highest level of education	
Elementary school or less	34.1% (n = 145)
Junior high school	26.1% (n = 111)
Senior high school	26.4% (n = 112)
College, university, or postgraduate	13.4% (n = 57)
% Religion	
Buddhist	48.7% (n = 206)
Taoist	31.9% (n = 135)
Other	19.4% (n = 82)
% Language (multiple answers possible)	
Mandarin	92.6% (n = 389)
Holo	80.2% (n = 337)
Hakka	30.2% (n = 127)
Other	3.1% (n = 13)
% Current occupation	
Employed	31.1% (n = 129)
Housework	51.1% (n = 212)
Unemployed	7.7% (n = 32)
Retired	10.1% (n = 42)
% Married/cohabited	81.0% (n = 341)
% With enough or sufficient economic resources	94.3% (n = 390)
% Current condition of health compared to people of similar age	
Excellent	16.1% (n = 69)
Very good	45.6% (n = 195)
Good	35.5% (n = 152)
Fair	2.6% (n = 11)
Poor	0.2% (n = 1)
Mean years lived in this community (±SD)	29.1 (±17.8)
% County of residence	
New Taipei	36.8% (n = 160)
Taoyuan	50.6% (n = 220)
Hsinchu	12.6% (n = 55)
% Serving a village rather than a city	51.0% (n = 222)
Median years (IQR)** spent working as a community health volunteer	4 (2.0, 6.3)
Median hours per week (IQR) volunteering	6.0 (4.0, 8.0)
Median number of people (IQR) serviced per week	30 (16, 60)
% CHPDC***	
Community based	52.6% (n = 229)
Public Health Centre based	11.3% (n = 49)
Hospital based	36.1% (n = 157)
% Volunteering job	
Fundamental	52.2% (n = 210)
Cadre	17.7% (n = 71)
Leader	15.7% (n = 63)
Chief (oversees volunteer services)	14.4% (n = 58)
% Time committed to other volunteering services	
One service	38.9% (n = 167)
Two services	25.9% (n = 111)
hree or more services	20.7% (n = 89)
Median years spent in all other voluntary services except community health promotion (IQR)	5 (3, 8)
Median total time in years spent in voluntary service (IQR)	8 (4.5, 14)
% Planning to stay on as a community health promotion volunteer	
Very unlikely	0.3% (n = 1)
Unlikely	0.5% (n = 2)
Neutral	4.1% (n = 16)
Likely	54.6% (n = 214)
Very likely	40.6% (n = 159)

*SD = standard deviation; Not all numbers add up to 435 because of missing values in the data, **IQR = Inter-quartile range; ***CHPDC = Community Health Promotion Development Centre; Not all numbers add up to 435 because of missing values in the data.

**Table 2:** The burden characteristics of the survey participants (N = 435) measured by the BCHV tool*

**Item**	**Mean**	SD**	**n**
1. I feel the low participation by the community residents in planned healthcare activities impacts on my role.	3.01	(± 0.98)	433
2. I feel I lack support from members of the community residents in carrying out my role	2.43	(± 0.98)	434
3. Misunderstanding by the community residents about the health service availability affects my role.	2.58	(± 1.03)	434
4. I feel the presence of fraudulent groups in Taiwan makes my role as a health care volunteer more difficult.	2.71	(± 1.20)	433
5. I feel the adaptation courses provided for volunteers did not adequately help me to conduct my role.	2.40	(± 1.10)	433
6. I feel the lack of volunteers makes my role more difficult.	2.86	(± 1.18)	431
7. I feel the benefits for volunteers are inadequate.	2.37	(± 1.33)	432
8. I feel my role is affected by missing or broken equipment.	2.48	(± 1.07)	433
9. I feel the difficulty associated with completing the required paper work impacts on my role.	2.62	(± 1.15)	432
10. I feel the frequent change of supervising strategies of the health government impacts on my role.	2.53	(± 1.22)	433
11. I feel that I have too many community activities to conduct to be effective.	2.17	(± 1.10)	432
12. I feel the courses provided for volunteers did not adequately prepare me for the health problems I manage.	2.56	(± 1.07)	426
13. I find it difficult to communicate among some of the community residents, such as immigrant residents, and this makes my role harder.	2.52	(± 1.05)	427
14. I am unclear about the expectations of my role.	1.70	(± 0.90)	427
15. I feel lack of communication and/or interaction with other volunteers makes my role more difficult.	1.60	(± 0.76)	428
16. I feel that the dangers associated with the health services and home care impact on my role.	1.59	(± 0.77)	428
17. I feel physically exhausted due to my volunteer role.	1.66	(± 0.82)	427
18. I feel the lack support of my family makes my role more difficult.	1.43	(± 0.75)	429
19. I feel it is difficult to arrange health schedules during holidays.	2.28	(± 0.97)	429
20. Overall, I feel the burdens associated with the volunteer’s role are heavy.	2.11	(± 0.87)	428
Overall mean sum of burdens	45.6	(± 11.5)	425
range	20-85		
Overall mean of single burdens	2.28	(± 0.58)	425

*BCHV = Burden on Community Health Volunteers; Each item was assessed from 1 = never, 2 = seldom, 3 = sometimes, 4 = often, 5 = always;

**SD = standard deviation.

The BCHV was tested for reliability and validity with CHVs in Taiwan. The internal consistency of the instrument satisfied the minimum recommended level of reliability for Cronbach’s coefficient alpha (0.823) with Cronbach’s alphas for test and re-test of 0.82 and 0.77 respectively (Gau, Buettner, Usher & Stewart, 2012). The content validity index (CVI) was 0.86.

### Ethical issues

Ethical approvals to conduct the study were sought and received from the University Human Research Ethics Committee (HREC) and the committee of the Institutional Review Board (IRB) of Chang Gung Medical Foundation in Taiwan. All participants in the study were voluntary.

### Statistical analysis

All data were entered into the Statistical Package for the Social Sciences [SPSS] (IBM SPSS version 18, Chicago, Illinois) for Windows. Descriptive statistics such as percentages, mean values, standard deviations and median values were calculated to describe the sample. This was followed by inferential statistics using Chi-square tests, and analysis of variance or the non-parametric Kruskal-Wallis test to determine whether the burden differed between the three groups of volunteers. Multivariate linear regression analysis was used to determine factors associated with the overall sum of the burden as the dependent variable.

## Results

### Study population

Demographic characteristics of the 435 participants who completed the study are described in Table 1. Most volunteers were female (84.3%), with a mean age of 59.1 ± 10.8 years, 39.8% had senior high school education or higher, 61.7% reported feeling “well” or “very well” considering their current health compared with people of a similar age, and 94.3% reported having “sufficient” or “enough” current economic resources.

The percentage of participants serving in the city and in village regions was almost the same (49% and 51%), and the median number of years working as a CHV was 4 years (Inter-quartile range [IQR] = 2.0, 6.3). The median number of hours per week spent volunteering was 6.0 (IQR = 4.0, 8.0), and the median number of people serviced per week was 30 (IQR = 16, 60).

### Burden experienced by surveyed community health volunteers

As shown in Table 2, the mean score for the work burden of the CHVs was 45.6 (Standard deviation [SD] ±11.5). The overall mean score per question was 2.28 (SD ±0.58), which indicated that the majority of participants experienced burden from ‘seldom to sometimes’. The top four burdens experienced were: item **1**; “I feel the low participation by the community residents in planned healthcare activities impacts on my role” (mean 3.01; SD ±0.98); item **6**; “I feel the lack of volunteers makes my role more difficult” (mean 2.86; SD ±1.18); item **4**; “I feel the presence of fraudulent groups in Taiwan makes my role as a community health volunteer more difficult” (mean 2.71; SD ±1.20); and, item **9**; “I feel the difficulty associated with completing the required paper work impacts on my role” (mean 2.62; SD ±1.15).

To explore significant independent factors associated with burden, a standard multiple linear regression analysis, adjusted for potential confounding variables, was performed (see Table 3). The final regression model was based on 310 participants. Nine demographic characteristics were statistically significant factors (model: *F* (11, 298) = 11.803, *p* < 0.001) related to burden associated with the CHV role. Together these demographic variables predicted 30.3% (R^2^ = .303) of the variance in the sum of the CHV burden. In addition, the final regression model was adjusted for the confounding effects of age (confounded marital status and health status) and employment status (confounded health status and time spend as a CHV) (Kleinbaum et al., 1982).

**Table 3:** Factors influencing burden of volunteer work in Taiwan (n = 310)

**Associates of the sum of all burdens**	**Standardised coefficient**	**Original coefficient**	**95%-CI**	**p-value**
**Service in rural area** (compared to service in the urban area)	−0.357	−8.084	−10.569, -5.599	P < 0.001
**Service 60 people or more per week** (compared to servicing less people)	0.375	10.030	12.804, 7.257	P < 0.001
**Service hours per week**				
3 to 11.9 hours per week	0.131	4.063	0.838, 7.287	P = 0.014
12 hours or more per week	0.144	4.759	0.874, 8.643	P = 0.017
**Work in Health Promotion Development Centre hospital based** (compared to community or public health centre based)	0.147	3.514	0.886, 6.142	P = 0.009
Time spent as a community health volunteer* (number of years)	−0.169	−0.597	−0.969, -0.225	P = 0.002
Total time spent in voluntary service overall** (number of years)	0.180	0.271	0.084, 0.459	P = 0.005
**Being a widow or widower** (compared to being single, unmarried, married, or divorced)	−0.117	−4.056	−7.629, -0.482	P = 0.026
**Health status in comparison to someone of similar age being average, not well or really unwell** (compared to well and very well)	0.147	3.449	1.192, 5.707	P = 0.003
**Model Summary**	R^2^ =0.303 *F*(11,298) = 11.803 (*p* < 0.001)			

*Coefficient pertains to every additional year spent as a community health volunteer.

**Coefficient pertains to every additional year spent in voluntary service overall.

Result of multiple linear regression analysis adjusted for the confounding effects of age and employment status.

Results of the linear regression analysis demonstrated that volunteers who served more than 60 people per week felt greater burden compared with people who served fewer people per week (*β* =10.030, *p* < 0*.*001). The sum of burden of participants who served 60 or more people per week was expected to be about 10.030 units higher then participants who served fewer people. Volunteers who served in a rural area felt on average less burdened than volunteers who served in an urban area (*β* = −8.084, p < 0.001) and volunteers who were a widow or widower felt less burdened than volunteers with another marital status (*β* = −4.056, *p* = 0*.*026). In addition, volunteers who perceived their own health as poor felt more burdened than those who perceived their health as good (*β* =3.449, p = 0.003). Moreover, the more hours a volunteer served per week, the higher the burden experienced. Volunteers who worked in a hospital based CHPDC experienced on average a higher level of burden than volunteers who worked in community or public health centre based CHPDCs (*β* =3.514, *p* = 0.009). The influence of the type of volunteer position on the sum of burden was not statistically significant in multivariate analysis: cadre (*p* = 0.814), leader (*p* = 0.378) and chief (*p* = 0.753) [compared to fundamental volunteers].

## Discussion

The study examined the relationships between a series of demographic variables and the role-related burden experienced by CHVs in Taiwan. CHVs complement the health workforce, and particularly assist paid community health workers. Understanding their level of role-related burden and finding ways to overcome it so that they continue volunteering in the future is critical as attrition from the volunteer role is high. The level of burden identified in the study was relatively low, which may be related to the rewarding social interactions associated with the role, the feelings of being useful to others, and the approval of volunteer work by society [18-20]. It is also possible that the volunteers with a higher level of burden than those who participated in the study had already left the service. In addition, those who participated in the study might not have felt as burdened by their role as those who did not participate, leading to an underestimation of burden. The results, however, indicate that those who are female, middle-aged, married and with a high school level education dominate the sample. This is consistent with other similar studies of volunteers [6,21,22] that found volunteers tended to be predominantly female, aged between 50 and 69 years, with a high school level education, and married. Other studies have found that ability to undertake volunteer activities depends upon the number and age of children, parents’ ages, marital status, employment status, and the nature of the volunteer work [23-25]. Furthermore, according to statistics from the Taiwan Ministry of Interior [6], the percentage of volunteers over the age of 65 years has risen from 8.81% in 2001 to 14.05% in 2010, indicating that healthy older persons in Taiwan are volunteering at higher rates than ever before.

Most prior research on CHVs has been undertaken using a qualitative approach and has therefore not attempted to measure the degree of difficulty that volunteers experience as they carry out their role. The results of this study reveal that CHVs in Taiwan experience a relatively low level of burden, similar to the results obtained by Murayama at el [26]. Volunteers give their time freely with the expectation of benefit to the local community [24,27]. However, the low participation by the community residents in planned healthcare activities, the presence of fraudulent groups in Taiwan (people involved in criminal activity who try to gain entry to homes for fraudulent purposes) that cause community members to be suspicious of newcomers including community health volunteers, and misunderstanding by the community residents about the health service availability, resulted in burden for the volunteers. Burden was also experienced because of insufficient numbers of volunteers available to carry out the required activities and individual volunteers having large numbers of people to serve. Previous studies have reported similar results [15,28,29].

Interestingly, the study found that those who perceived their current health condition as good (97.8%) experienced less role-related burden. A study by Erlinghagen and Hank [30] found a strong relationship between health status and the rate of engagement in voluntary work, with much lower activity rates among those who perceived their current health status as ‘fair or worse’ (0. 6%) than those who reported ‘good or better’ health (12%). Wilson [24] claims that volunteering is not only able to improve one’s health, but also is able to preserve good health and keep healthy volunteers healthy.

The present research adds to the current knowledge base by enhancing understanding of burden experienced by volunteers from different facilities: community, hospital and public health centres. The burden experienced was statistically significant between groups, with hospital based volunteers experiencing higher levels of burden than the community or public health centre based CHVs, when adjusted for the confounding effects of age and employment status. One explanation for these differing levels of burden may be that volunteers at hospital based centres serve clients who seek medical support from broader regions around the hospital and may not be local residents. The clients may also have more serious health issues. Our findings also reveal that volunteers who served in a rural area experienced a lower level of burden than volunteers who served in an urban area. This finding is in direct contrast to the finding of Freeman at el. [31], who found that rural volunteers had high levels of attrition due to high levels of burden and difficulty in meeting continuing education requirements. The difference between the study contexts and different sampling techniques may account for the disparity. However, as large numbers of people live in urban areas, it could be argued that there is a greater demand for health promotion and monitoring activity in urban areas.

Most studies of community volunteers argue that when volunteers experience greater satisfaction and perceive more relative benefits from their role, they are likely to remain actively involved for longer periods of time [16,32,33]. Unfortunately, this study shows that the longer a person has volunteered overall the more s/he felt burdened. This result may indicate that more experienced volunteers are expected to take on more responsibility, manage more people, and oversee the work of other volunteers, which may increase their level of burden.

This study had several limitations in relation to methodological and sampling issues. First, the study sample was drawn only from community health services in the Northern regions of Taiwan. CHVs in other areas may have different results. In addition, the demographics section did not ask the volunteers about the number and ages of their children, and the age of their parents. Further, the design was cross-sectional rather than longitudinal, so it only offers a snapshot in time that does not allow causative conclusions to be drawn. In addition, because volunteers who had left the service were not interviewed, survivor bias could be present.

## Conclusions

CHVs have an important role that adds significantly to the health workforce and provides many services that would otherwise be unavailable because of funding cutbacks. In this study, 435 participants completed the BCHV questionnaire. The overall mean of the volunteer burden was low on average. However, several items of the BCHV revealed areas of higher burden that need to be addressed in the future to prevent further attrition of community volunteers. The results of the multivariate adjusted regression analysis revealed significant differences in overall volunteer burden depending on the number of people served on average per week, marital status, and whether or not volunteers worked as health promotion volunteers. Volunteers who served many hours, and who perceived their own health as not good, felt more burdened than other volunteers.

## Abbreviations

CHPDC: Community Health Promotion Development Centre; CHVs: Community Health Volunteers; CVI: Content Validity Index; HREC: Human Research Ethics Committee; IQR: Inter-quartile Range; IRB: Institutional Review Board; SD: Standard Deviation.

## Competing interests

The authors declare that they have no competing interests.

## Authors’ contributions

YMG, KU, and LS developed the study design and managed the project. YMG collected all data. YMG, KU and PB conducted the data entry and analysis. All authors contributed to writing and reviewing the final paper.

## Pre-publication history

The pre-publication history for this paper can be accessed here:

http://www.biomedcentral.com/1471-2458/13/491/prepub

## References

[B1] Chandra RK Whole health: a prescription for the new millennium Nutr Res 2001 21 1 8 10.1016/S0271-5317(00)00299-2

[B2] Fried LP Freedman M Endres TE Wasik B Building communities that promote successful aging West J Med 1997 167 216 219 9348750 PMC1304534

[B3] Cheung CK Ma SK How older residents benefit from the management of volunteer service Adm Soc Work 2010 34 241 258 10.1080/03643107.2010.480929

[B4] Lamb C Outlook the future challenges for volunteers, major organisations and the international community Refugee Survey Quarterly 2006 25 173 178 10.1093/rsq/hdi0187

[B5] Lewin S Munabi-Babigumira S Glenton C Daniels K Bosch-Capblanch X van Wyk BE Odgaard-Jensen J Johansen M Aja GN Zwarenstein M Scheel IB Lay health workers in primary and community health care for maternal and child health and the management of infectious diseases Cochrane Database Syst Rev 2010 3 1 177 10.1002/14651858.CD004015.pub3 PMC6485809 20238326

[B6] The current services of the volunteers in Taiwanhttp://vol.moi.gov.tw/vol/index.jsp

[B7] Bureau of Health Promotion DoH Work manual for the construction of community health (Chinese) 2011 Taiwan: Bureau of Health Promotion, D.o.H., R. O. C

[B8] Chen CM Yang SC Sustainable strategies for healthy community center operations Evidence of Nursing (Taiwan) 2006 2 250 258

[B9] Couto RA Promoting health at the grass roots Health Aff 1990 9 144 151 10.1377/hlthaff.9.2.144 2365255

[B10] Huang CL Wang HH Community health development: what is it? Int Nurs Rev 2005 52 13 17 10.1111/j.1466-7657.2004.00259.x 15725271

[B11] Bureau of Health Promotion DoH Taiwan Public Health Report 2006 Taiwan, Taipei: Bureau of Health Promotion, Department of Health Bureau of Health Promotion DoH, R. O. C

[B12] Gaugler JE Roth DL Haley WE Mittelman MS Can counseling and support reduce burden and depressive symptoms in caregivers of people with Alzheimer's disease during the transition to institutionalization? Results from the New York University caregiver intervention study J Am Geriatr Soc 2008 56 421 428 10.1111/j.1532-5415.2007.01593.x 18179495 PMC2700042

[B13] Papastavrou E Kalokerinou A Papacostas SS Tsangari H Sourtzi P Caring for a relative with dementia: family caregiver burden J Adv Nurs 2007 58 446 457 10.1111/j.1365-2648.2007.04250.x 17442030

[B14] Whitlatch CJ Zarit SH von Eye A Efficacy of interventions with caregivers: a reanalysis Gerontologist 1991 31 9 14 10.1093/geront/31.1.9 2007480

[B15] Akintola O Defying all odds: coping with the challenges of volunteer caregiving for patients with AIDS in South Africa J Adv Nurs 2008 63 357 365 10.1111/j.1365-2648.2008.04704.x 18727763

[B16] Garland D Myers D Wolfer T Protestant Christian volunteers in Community Social Service Programs: what motivates, challenges, and sustains their service Adm Soc Work 2009 33 23 39 10.1080/03643100802508627

[B17] Kang'ethe SM Challenges impacting on the quality of care to persons living with HIV/AIDS and other terminal illnesses with reference to Kanye community home-based care programme Sahara J-J Soc Asp H 2009 6 24 32 10.1080/17290376.2009.9724926 19399313

[B18] Akintola O What motivates people to volunteer? The case of volunteer AIDS caregivers in faith-based organizations in KwaZulu-Natal, South Africa Health Policy Plan 2011 26 53 62 10.1093/heapol/czq019 20511348

[B19] Clary EG Snyder M Ridge RD Copeland J Stukas AA Haugen J Miene P Understanding and assessing the motivations of volunteers: a functional approach J Pers Soc Psychol 1998 74 1516 1530 9654757 10.1037//0022-3514.74.6.1516

[B20] O'Brien L Townsend M Ebden M 'Doing something positive': volunteers' experiences of the well-being benefits derived from practical conservation activities in nature Voluntas 2010 21 525 545 10.1007/s11266-010-9149-1

[B21] Cheng L The effectiveness of health building project on changing volunteers’ health behaviors in the communities of Southern Taiwan 2007 Kaohsiung: Kaohsiung Medical University

[B22] Lee C Dobson AJ Brown WJ Bryson L Byles J Warner-Smith P Young AF Cohort profile: the Australian longitudinal study on women's health Int J Epidemiol 2005 34 1093 1098 10.1093/ije/dyi098 15894591

[B23] Wardell F Lishman J Whalley LJ Who volunteers? Brit J Soc Work 2000 30 227 248 10.1093/bjsw/30.2.227

[B24] Wilson J Volunteering Annu Rev Sociol 2000 26 215 240 10.1146/annurev.soc.26.1.215

[B25] Wilson J Musick MA Attachment to volunteering Sociological Forum 1999 14 243 272 10.1023/A:1021466712273

[B26] Murayama H Taguchi A Murashima S Development of satisfaction and burden scales for community activities of health promotion volunteers Nippon Koshu Eisei Zasshi 2006 53 875 883 17274385

[B27] Thoits PA Hewitt LN Volunteer work and well-being J Health Soc Behav 2001 42 115 131 10.2307/3090173 11467248

[B28] Kironde S Bajunirwe F Lay workers in directly observed treatment (DOT) programmes for tuberculosis in high burden settings: Should they be paid? A review of behavioural perspectives Afr Health Sci 2002 2 73 78 12789106 PMC2141572

[B29] Shaibu S Community home-based care in a rural village: challenges and strategies J Transcult Nurs 2006 17 89 94 10.1177/1043659605281974 16410441

[B30] Erlinghagen M Hank K The participation of older Europeans in volunteer work Ageing and Soc 2006 26 567 585 10.1017/S0144686X06004818

[B31] Freeman VA Slifkin RT Patterson PD Recruitment and retention in rural and urban EMS: results from a national survey of local EMS directors J Public Health Manag Pract 2009 15 246 252 19363405 10.1097/PHH.0b013e3181a117fc

[B32] Cuthill M Warburton J A conceptual framework for volunteer management in local government Urban Pol Res 2005 23 109 122 10.1080/0811114042000335269

[B33] Omoto AM Snyder M Sustained helping without obligation: motivation, longevity of service, and perceived attitude change among AIDS volunteers J Pers Soc Psychol 1995 68 671 686 7738770 10.1037//0022-3514.68.4.671

